# Solvent Free Upgrading of 5-Hydroxymethylfurfural (HMF) with Levulinic Acid to HMF Levulinate Using Tin Exchanged Tungstophosphoric Acid Supported on K-10 Catalyst

**DOI:** 10.1021/acsorginorgau.2c00027

**Published:** 2022-10-03

**Authors:** Manishkumar S. Tiwari, Dipti Wagh, Jennifer Sarah Dicks, John Keogh, Michela Ansaldi, Vivek V. Ranade, Haresh G. Manyar

**Affiliations:** † Theoretical and Applied Catalysis Research Cluster, School of Chemistry and Chemical Engineering, Queen’s University Belfast, David-Keir Building, Stranmillis Road, BelfastBT9 5AG, U.K.; ‡ Department of chemical Engineering, Mukesh Patel School of Technology Management and Engineering, SVKM’s NMIMS University, Mumbai, India400065

**Keywords:** Levulinic acid, HMF levulinate, Biofuels, Heteropoly acids, Tin exchanged tungstophosphoric acid, Montmorillonite K10

## Abstract

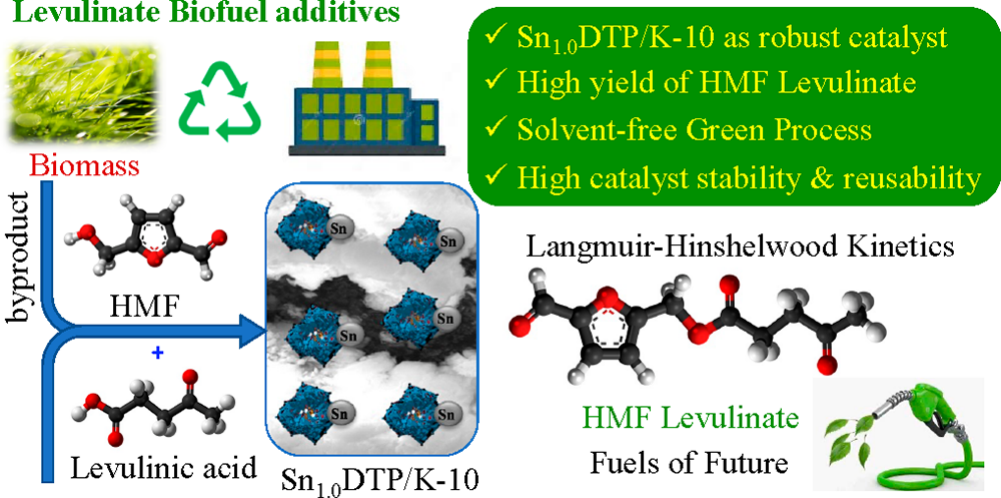

The manufacture of high-value products from biomass derived platform chemicals is becoming an integral part of the biorefinery industry. In this study, we demonstrate a green catalytic process using solvent free conditions for the synthesis of hydroxymethylfurfural (HMF) levulinate from HMF and levulinic acid (LA) over tin exchanged tungstophosphoric acid (DTP) supported on K-10 (montmorillonite K-10 clay) as the catalyst. The structural properties of solid acid catalysts were characterized by using XRD, FT-IR, UV–vis, titration, and SEM techniques. Partial exchange of the H^+^ of DTP with Sn (*x* = 1) resulted in enhanced acidity of the catalyst and showed an increase in the catalytic activity as compared to the unsubstituted DTP/K-10 as the catalyst. The effects of different reaction parameters were studied and optimized to get high yields of HMF levulinate. The kinetic model was developed by considering the Langmuir–Hinshelwood–Hougen–Watson (LHHW) mechanism, and the activation energy was calculated to be 41.2 kJ mol^–1^. The prepared catalysts were easily recycled up to four times without any noticeable loss of activity, and hot filtration test indicated the heterogeneous nature of the catalytic activity. The overall process is environmentally benign and suitable for easy scale up.

## 1 Introduction

Continued depletion of the available fossil fuel resources, coupled with increasing energy demand and rising greenhouse gases, has led to our quest for alternate sources of renewable energy and chemical feedstocks.^1^ Biomass has emerged as one of the prominent alternative feedstocks for the production of sustainable fuels and useful platform chemicals.^2,3^ Lignocellulosic biomass is being extensively explored as a feedstock for the synthesis of several high-value chemicals such as ethanol, furfural, lactic acid, 5-hydroxymethylfurfural (HMF), and levulinic acid (LA).^4−6^ HMF and LA have been recognized by the U.S. Department of Energy (DOE) as one of the top 12 platform chemicals derived from biomass,^7−9^ which can be further transformed to a variety of renewable chemicals such as furans, furanic acids, and esters. Among HMF derived chemicals, HMF esters are very versatile with numerous applications in the chemical industry. HMF esters can be synthesized by condensation between HMF and bioderived acids such as lactic acid, pyruvic acid, formic acid, acetic acid, and levulinic acid. HMF esters are valuable intermediates in fine chemical, pharmaceutical, and biorefinery industries with numerous applications in the production of fungicides and active pharmaceutical ingredients (APIs), and as fuel additives and surfactants.^10−12^ Esterification reactions are usually performed using either homogeneous or heterogeneous catalysts, and particularly in the case of HMF as the reactant the use of homogeneous catalysts not only results in separation issues but also leads to byproduct formation due to various side reactions such as self-etherification of HMF or polymerization of HMF.^2,12,13^ Hence, for esterification of biomass derived compounds, various heterogeneous acid catalysts such as acidic resins, sulfonic acid functionalized on SBA-15, or bifunctional ionic liquids are desired catalysts.^10,11,14−17^ The high cost of catalysts, reusability, and unwanted byproduct formation are the main drawbacks of the current processes, which need to be overcome to develop the desired economically viable and efficient processes for HMF ester synthesis. In particular, for HMF esterification with LA, literature is scarce with only a few reports published so far, using enzymes^10^ and ionic liquids.^11^ Hence, an efficient and sustainable catalytic method for synthesizing HMF levulinate is certainly needed for sustainable production at a large scale. Further, the current studies were performed under solventless conditions, which improves the green quotient of the process including several advantages, such as ease of separation of catalyst and products, no waste generation due to solvent recovery or disposal, low cost of operation, enhanced activity, etc.

**Scheme 1:**
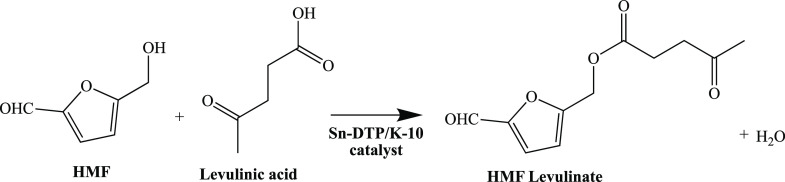
Sn-DTP/K-10 Catalyzed Esterification of HMF and LA to HMF Levulinate

Heteropoly acids supported on montmorillonite clay K-10 have been established as highly efficient and selective catalysts in a variety of reactions.^5,18^ Immobilizing heteropoly acids such as tungstophosphoric acid (DTP) on various supports has not only resulted in enhanced activity but also helped to overcome the leaching issue.^6,19−21^ However, the leaching issue is not completely avoidable; hence, exchanging the protons of heteropoly acid with metal ions to make an insoluble salt is often a preferred solution. In this context, various metal ions such as Cs^+^, Al^3+^, Ag^+^, and Hf ^4+^ have been exchanged with protons to make insoluble salts of DTP. The exchange of proton also increases the overall acidity of the catalysts.^22−24^ Unsupported tin exchanged DTP salts were prepared by Lingaiah et al., which showed high activity in benzylation of arenes.^25^ However, unsupported catalysts are difficult to recover and recycle due to the loss of catalytic activity upon agglomeration. To immobilize the tin exchanged DTP salts, acidic clays such as montmorillonite K-10 are most suitable support due to good surface area, surface acidity, and excellent dispersion of Keggin ions to improve the catalytic activity vis-à-vis unsupported HPAs. In the present study, tin exchanged tungstophosphoric acid supported on K-10 catalyst was synthesized using the incipient wetness technique and characterized by using different techniques. Synthesized catalyst was used in the catalytic esterification of HMF and LA to produce HMF levulinate (Scheme 1). Various reaction parameters were optimized to maximize the yield, and a mathematical model for kinetic analysis was proposed to provide a rational basis for the design of the process for production of HMF levulinate esters.

## 2 Experimental Section

### 2.1 Materials and Methods

All chemicals were procured and used without any further purification. HMF, LA, tungstophosphoric acid hydrated, tin chloride, and montmorillonite K-10 clay were purchased from Sigma-Aldrich, UK. All catalysts were prepared and tested in house.

### 2.2 Catalyst Synthesis

The supported catalysts 20% (w/w) DTP/K-10 and 20% (w/w) Sn_1_DTP/K-10 were prepared by the incipient wetness technique. An amount of 5 g of K-10 was kept for drying at 120 °C for 12 h. To prepare 2 g of 20% w/w Sn_1_DTP/K-10 catalyst, 1.6 g of dried K-10 was impregnated with 2 mL of alcoholic solution of SnCl_2_ and dried for 5 h at 120 °C to get the free-flowing solid. The impregnated solid was then used for the second impregnation of DTP using the alcoholic solution and dried at 120 °C for 12 h followed by calcination at 300 °C for 3 h. Then 20% w/w DTP/K-10 was also prepared by using a similar incipient wetness technique as described above, except without the SnCl_2_ impregnation step.

### 2.3 Catalyst Characterization

Solid samples were characterized by different techniques, and details are given herein. XRD was done using a PANalytical X-Pert Pro MPD diffractometer provided with Ni filtered Cu Kα radiation (1.5405 Å) in the range of 5–80° and at a step size of 0.016°. FT-IR analysis was performed using a PerkinElmer Fourier transform infrared spectrometer. The thin waferlike samples were prepared by pressing 1 mg of catalyst in 100 mg of dried KBr. UV–vis spectra were recorded using a Shimadzu UV-1280 03540 spectrometer for all catalysts prepared in house. SEM images were recorded using a FEI Quanta FEG 250 scanning electron microscope for different samples. The samples were dried, sputter coated, and then scanned via SEM at various magnifications. Using acid–base titration, the acidity of the prepared catalyst samples was measured using titration.^26,27^ In 25 mL of 0.1 M NaOH solution, 100 mg of solid catalyst was stirred for 6 h and then titrated with 0.1 M HCl solution to get the acidity of the samples.

### 2.4 Catalyst Activity Testing

All reactions were performed in a cylindrical glass reactor of 30 mL volume, equipped with baffles and agitated with a 4-bladed stirrer placed in an oil bath. The calculated amount of HMF and LA was placed in the reactor and stirred for a few minutes to get the homogeneous solution. Once the reaction mixture reached the desired temperature, an initial zero minute sample was taken and the catalyst was added and stirring was started. The reaction mixture was maintained under isothermal conditions until the reaction was completed, usually within 2 h. The reaction mixture was agitated above 800 rpm with the stirrer speed optimized to ensure removal of external mass transfer limitations. The samples were taken at regular intervals for the analysis. The withdrawn sample was then centrifuged to remove any solid particle and analyzed by gas chromatography with a FID detector and HP-5 capillary column.

## 3 Results and Discussion

### 3.1 Catalyst Characterization

**Figure 1:**
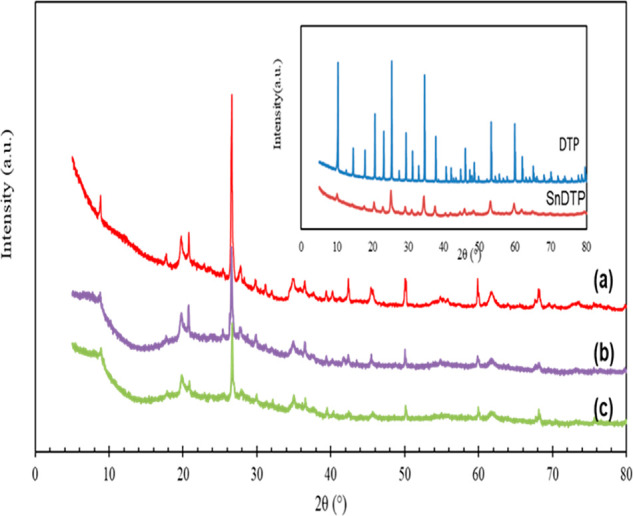
X-ray diffraction patterns of different catalysts: (a) K-10 clay, (b) DTP/K-10, and (c) Sn_1_DTP/K-10.

**Figure 2:**
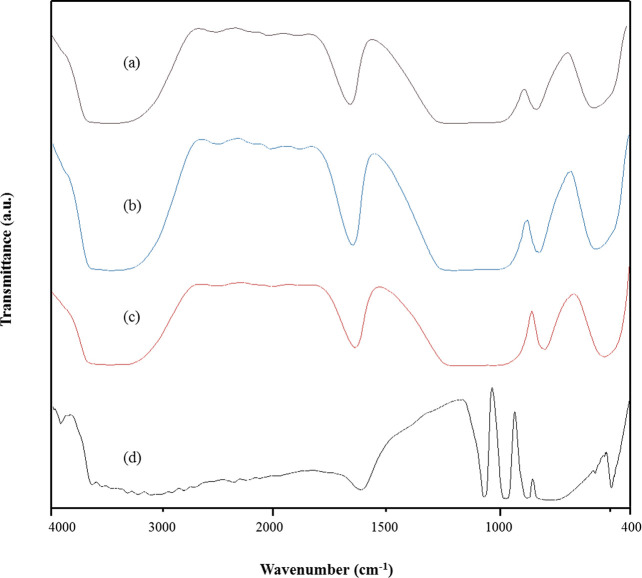
FT-IR spectra of catalysts: (a) Sn_1_-DTP/K-10, (b) DTP/K-10, (c) K-10 clay, and (d) DTP.

**Figure 3:**
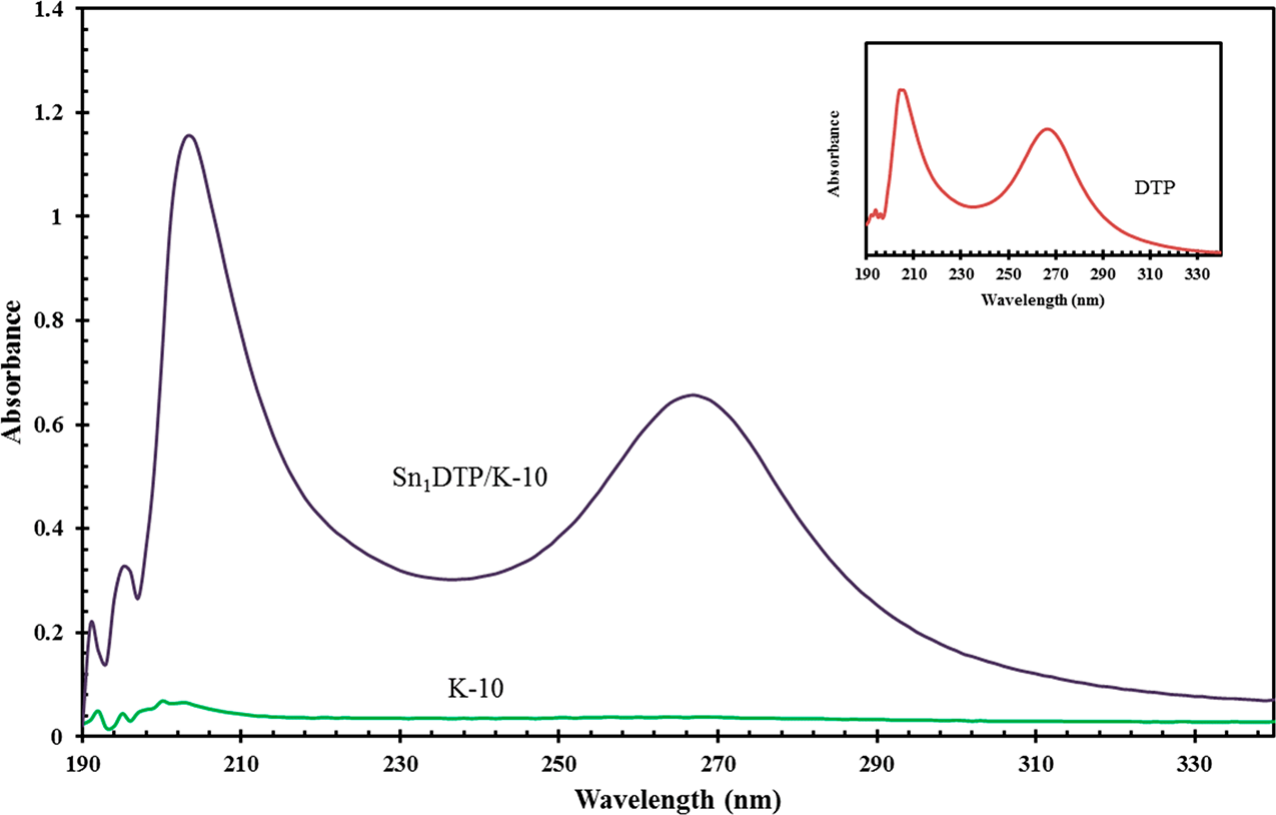
UV–vis spectra of catalysts DTP, Sn_1_-DTP/K-10, and K-10 clay.

XRD patterns of K-10, DTP/K-10, and Sn_1_DTP/K-10 are provided in Figure 1. The XRD pattern of K-10 shows peaks at 20° and 35° relating to the 110 and 105 facets of K-10 along with a peak at 26° corresponding to the K-10 impurity.^5^ The overall spectra thus confirm the crystalline nature of K-10 as reported earlier.^18^ DTP is highly crystalline as evident from the XRD patterns shown in the inset. After exchanging the protons with tin, the peak intensity decreased slightly (Figure 1b and c); however, still the presence of sharp peaks confirmed the crystalline nature of the supported Sn-DTP salts. The FT-IR spectra of Sn_1_DTP/K-10, DTP/K-10, K-10, and DTP are shown in the Figure 2. The characteristic peaks of the Keggin structure of DTP can be seen at 789 cm^–1^ (edge sharing W–O–W), 890 cm^–1^ (corner W–O–W), 986 cm^–1^ (terminal W=O), and 1080 cm^–1^ (P–O bonds).^18,20^ The FT-IR spectra of K-10 show a broad hump between 750 and 1100 cm^–1^. The overall spectra of Sn_1_DTP/K-10 are similar to K-10, which indicates that there was no bond formation or interaction between the DTP molecules with K-10. To confirm the Keggin structure of Sn_1_DTP supported on K-10, UV–vis data of the prepared catalyst was compared with that of K-10 (Figure 3). As shown in Figure 3 (inset), DTP shows two characteristic absorption bands related to the Keggin structure at 204 and 265 nm corresponding to the charge transfer for terminal oxygen to tungsten and the charge transfer from bridge oxygen to metallic tungsten, respectively.^28^ K-10 has no absorption peak,^28^ while the catalyst Sn_1_DTP/K-10 shows peaks at 204 and 265 nm which confirms the retention of the Keggin structure. The acidity of catalyst was determined by using the titration method.^27^ The acidity of prepared catalyst was found to be high and in the following order: bentonite clay (least) < K-10 < 20% w/w DTP/K-10 < 20% w/w Sn_1_DTP/K-10 (highest). The K-10 and bentonite clays are acid treated clays, and the acidity further increases with the impregnation of DTP and Sn_1_DTP. This showed that exchanging the H^+^ of DTP with Sn resulted in the increase in the acidity, which could be due to the availability of free ions which is responsible for the increase in acidity as reported earlier.^22,23^

**Figure 4:**
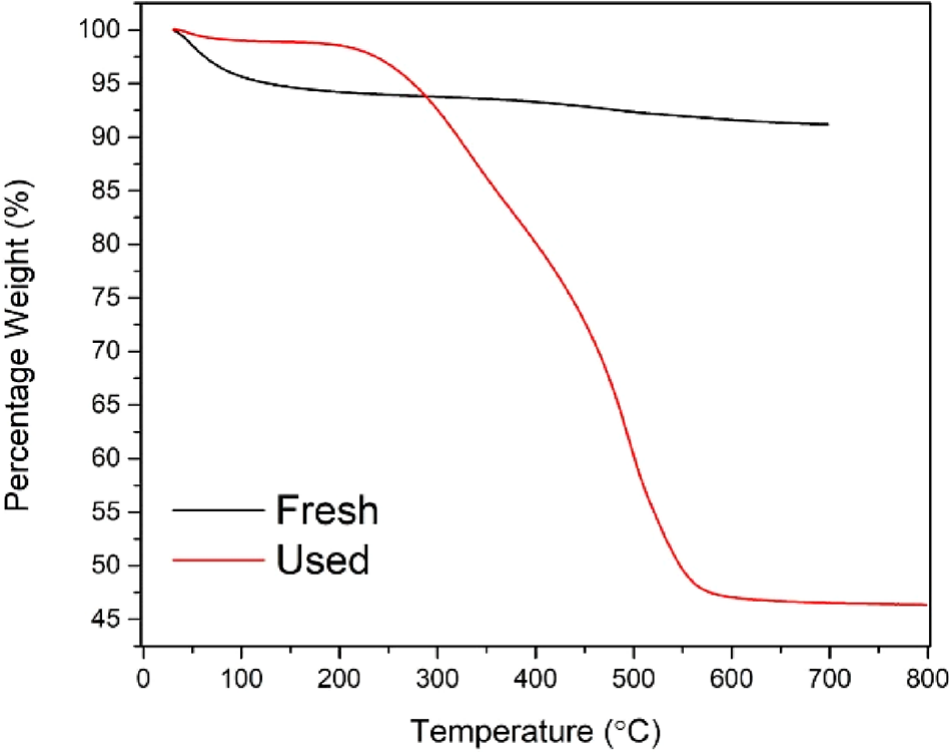
TGA of fresh Sn_1_-DTP/K-10 and used Sn_1_-DTP/K-10 catalysts.

**Figure 5:**
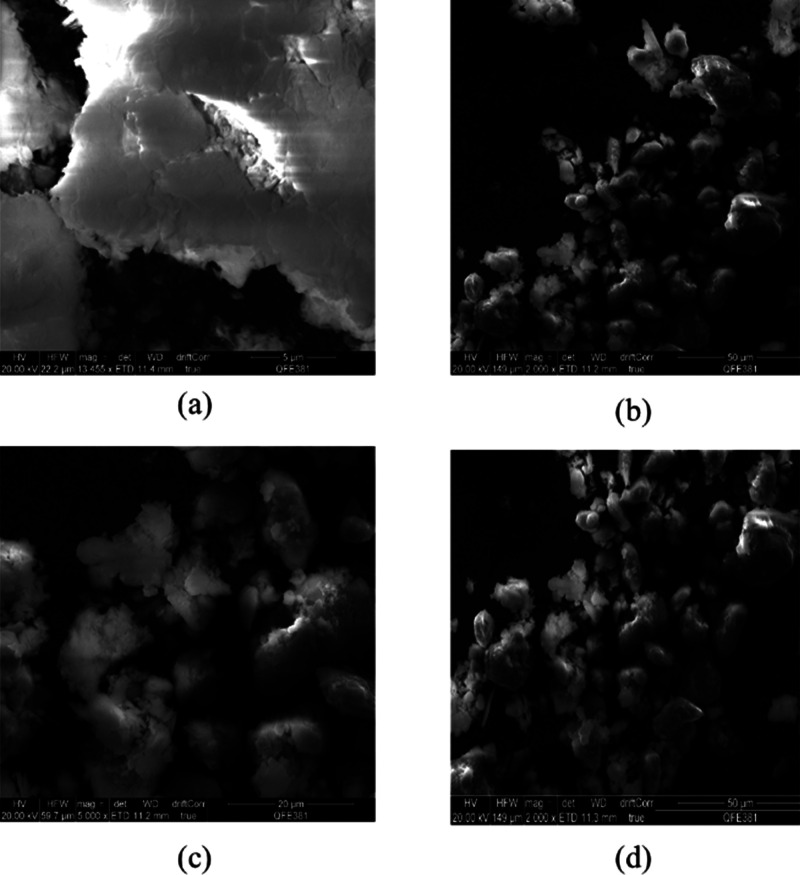
SEM images of (a, b) fresh 20% w/w Sn_1_-DTP/K-10 catalyst and (c, d) used 20% w/w Sn_1_-DTP/K-10 catalyst.

TGA analysis of the fresh and used catalyst samples helps to evaluate the catalyst regeneration temperature required to remove all adsorbed organics from the surface of the used catalyst. As shown in Figure 4, the first weight loss for fresh catalyst below 100 °C may be attributed to the removal of surface adsorbed water. Significant weight loss between 300 and 600 °C was noticed for the reused catalyst, which could be due to the desorption of adsorbed organics from the reaction. The SEM images show the formation of a uniform size of particles with irregular morphology (Figure 5). The fresh and used catalysts showed similar morphology, which indicated the structural stability of tin exchanged Keggin anions under experimental conditions.

### 3.2 Catalytic Activity in the Esterification of HMF and LA

The prepared catalysts 20% w/w Sn_1_DTP/K-10 and 20% w/w DTP/K10 alongside K-10 and bentonite clays were screened to compare the efficacy of the catalysts in the esterification of HMF and LA to HMF levulinate (Table 1). The solventless reaction between HMF and LA was conducted at 80 °C and 1000 rpm of agitation. The results were compared after 2 h in terms of conversion of HMF. The efficacy of the catalysts was in following order: bentonite clay (least) < K-10 < 20% w/w DTP/K-10 < 20% w/w Sn_1_-DTP/K-10 (highest). This also can be correlated with the acidity of catalysts. As 20% w/w Sn_1_DTP/K-10 was found to be the most active catalyst, further optimization of reaction parameters was performed by using 20% w/w Sn_1_DTP/K-10 as the catalyst.

**Table 1:** Effect of Various Catalysts on the Conversion of HMF and Correlation with Overall Acidity of Different Catalysts^a^

entry	catalyst	acidity (mmol g^-1^)	HMF conversion (%)
1	bentonite clay	0.24	12.6
2	montmorillonite K-10 clay	0.82	25.2
3	20% w/w DTP/K-10	1.45	47.5
4	20% w/w Sn_1_DTP/K-10	2.21	58.1

a Reaction conditions: HMF: 8.25 mmol, LA: 41.25 mmol, catalyst: 0.2 g, temperature: 80 °C, speed of agitation: 1000 rpm, reaction time: 120 min.

### 3.3 Optimization of Reaction Parameters

**Figure 6:**
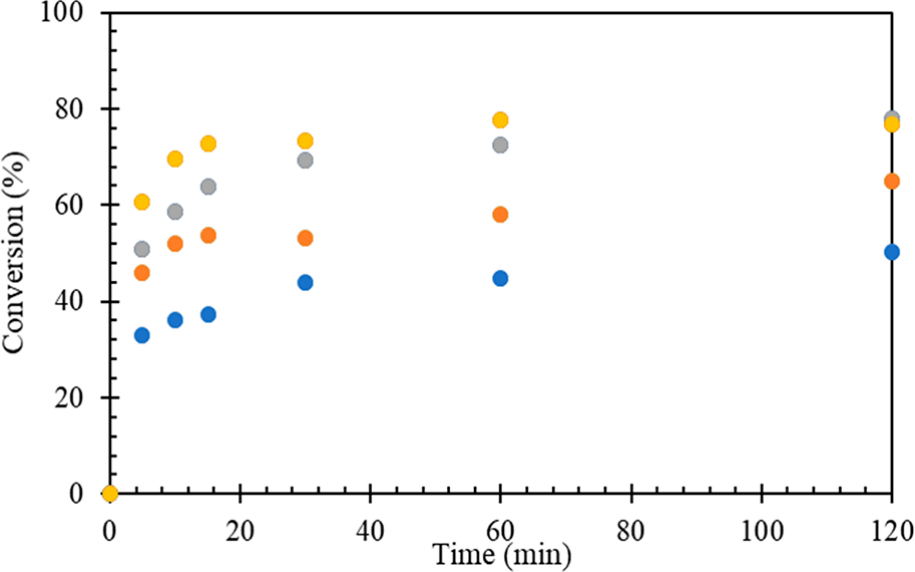
Effect of catalyst loading on the conversion of HMF, HMF: 8.25 mmol, LA: 41.25 mmol, 20% w/w Sn_1_DTP/K-10 catalyst, temperature: 80 °C, speed of agitation: 1000 rpm, reaction time: 120 min. Catalyst weight: (blue solid circle) 0.05 g, (orange solid circle) 0.1 g, (gray solid circle) 0.2 g, and (yellow solid circle) 0.3 g.

**Figure 7:**
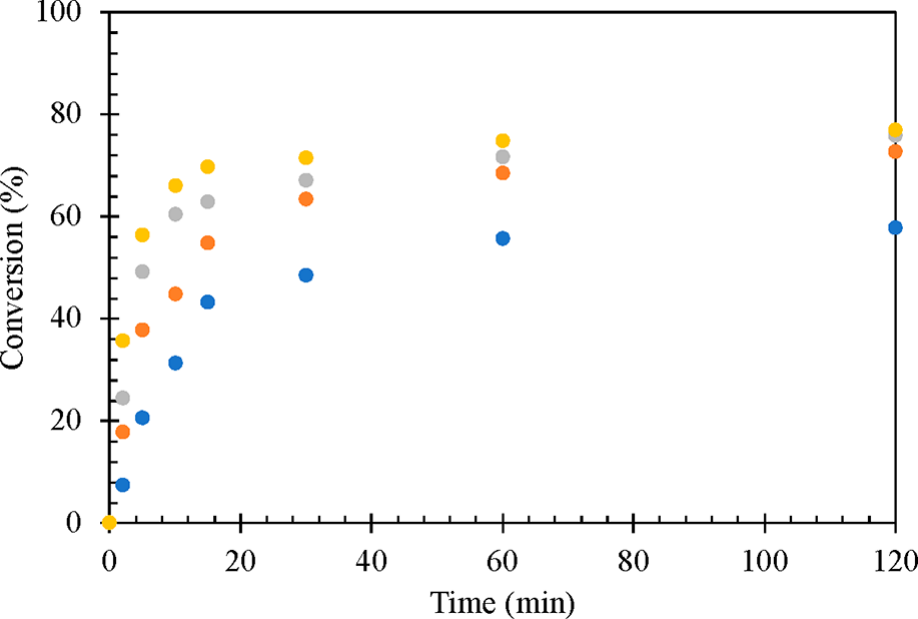
Effect of mole ratio of HMF to LA on the conversion of HMF: 20% w/w Sn_1_DTP/K-10 catalyst, catalyst weight: 0.2 g, temperature: 80 °C, speed of agitation: 1000 rpm, reaction time: 120 min. (Blue solid circle) 1:1, (orange solid circle) 1:3, (gray solid circle) 1:5, and (yellow solid circle) 1:7.

**Figure 8:**
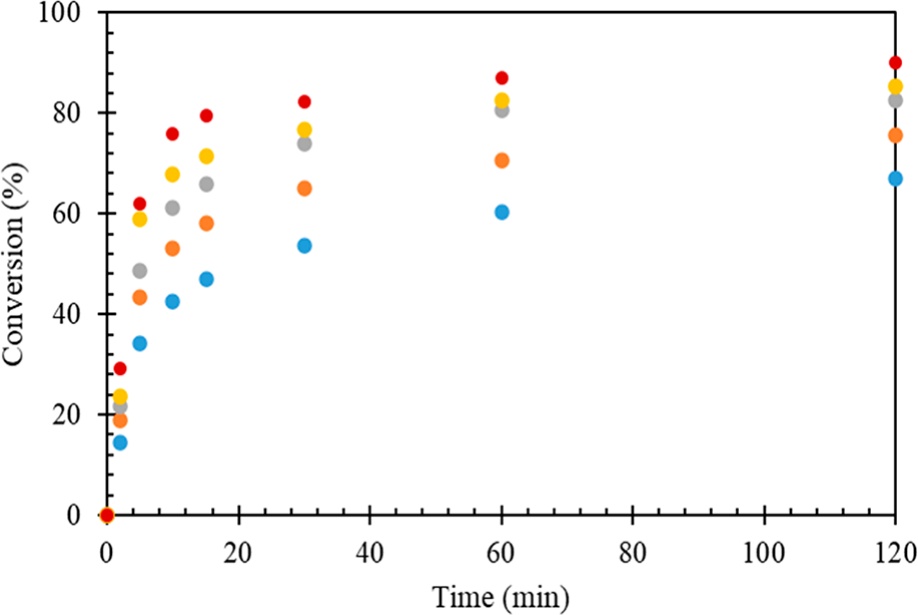
Effect of temperature on conversion of HMF: HMF: 8.25 mmol, LA: 41.25 mmol, 20% w/w Sn_1_DTP/K-10 catalyst, catalyst weight: 0.2 g, speed of agitation: 1000 rpm, reaction time: 120 min. (Blue solid circle) 70 °C, (orange solid circle) 80 °C, (gray solid circle) 90 °C, (yellow solid circle) 100 °C, and (red solid circle) 110 °C.

It was desirable to study the effect of various reaction parameters such as the catalyst amount, mole ratio of HMF to LA, and reaction temperature to optimize the catalytic esterification of HMF and LA to produce HMF levulinate. The effect of catalyst loading was studied using 20% w/w Sn_1_-DTP/K-10 as the catalyst, over a range of 0.04–0.25 mol % HMF. The percent conversion of HMF increased with an increase in the catalyst loading (Figure 6). The effect of mole ratio of HMF to LA was studied over the range of 1:1 to 1:7 by using 0.17 mol % 20% w/w Sn_1_-DTP/K-10 as catalyst while keeping other reaction parameters constant (Figure 7). The increase in the HMF:LA mole ratio resulted in an excess amount of LA, which acted as a solvent and helped to solubilize the formed product and free the active sites for further reaction. At 1:7 mol ratio of HMF:LA, a yield of 78.7% HMF levulinate was achieved. The final yield of product at 1:5 and 1:7 mol ratio of HMF:LA after 120 min was found to be almost same; hence, 1:5 mol ratio of HMF:LA was taken as optimum for further study. The increase in temperature results in an increase in the rate of reaction and a corresponding increase in the conversion of HMF (Figure 8). This also indicated that the presence of diffusion resistance is unlikely.

### 3.4 Development of Kinetic Model

Under the optimized conditions, the rate increases with an increase in temperature, indicating that the reaction is taking place under the kinetic regime. To find the activation energy of reaction and kinetic model to represent the reaction, the Langmuir–Hinshelwood–Hougen–Watson (LHHW) mechanism of adsorption of both reactants was assumed. The adsorption of both reactant and desorption steps was assumed to be very fast with the surface reaction as the controlling step.

Consider A (HMF), B (LA), C (HMF levulinate), and D (water). The adsorption of HMF and LA to the catalytic surface S is given by
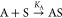
1
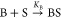
2The surface reaction of adsorbed species AS and BS gives the intermediate CS as
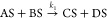
3The desorption step of different products can be written as
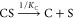
4
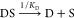
5assuming the reaction between adsorbed species is slower than the adsorption and desorption steps and hence considered as a rate-determining step. This means all other steps will be in equilibrium and the intermediate concentration can be written as
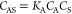
6a
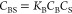
6b
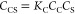
6c
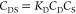
6dThe rate equation for the slowest steps can be written as
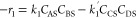
7The total catalytic site balance can be given as
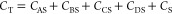
8From eqs 6 and 8, we have
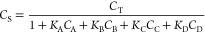
9From eqs 6, 7, and 9, we have
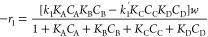
10where *w* is the total catalyst weight and equivalent to *C*_T_.

For the initial rate data analysis, the above reaction can be written as
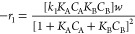
11

**Figure 9:**
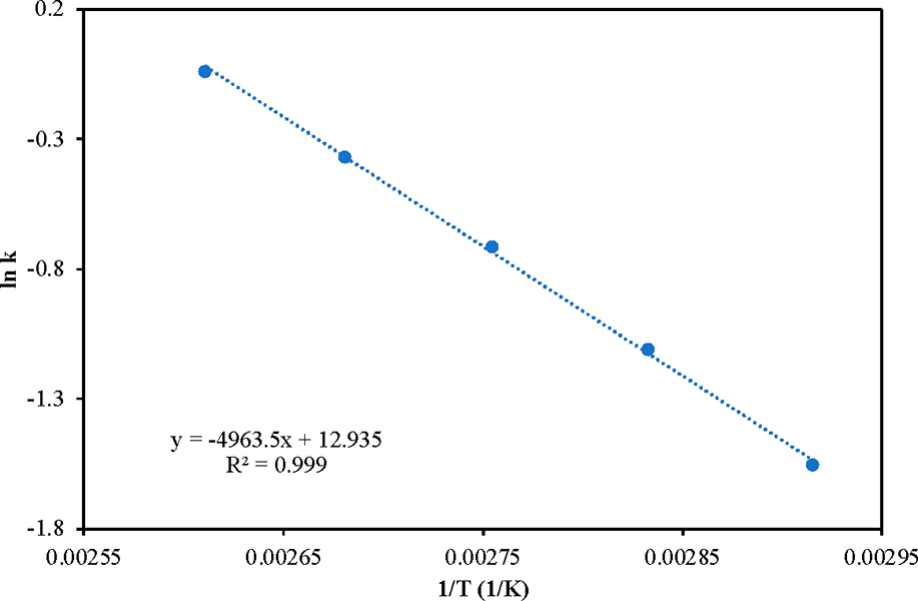
Arrhenius Plot for upgrading HMF to HMF levulinate using 20% w/w Sn_1_DTP/K-10 catalyst.

The obtained experimental results for temperature study were used to calculate the different constant using Solver, and the results are tabulated in Table 2. The rate constant values were used to make the Arrhenius plot (Figure 9), and the activation energy was calculated as 41.2 kJ/mol. The observed activation energy is larger than the activation energy of diffusion in liquids (12–21 kJ/mol), thus indicating the absence of external mass transfer and intraparticle diffusion resistances.^29,30^

**Table 2:** Kinetics Parameters for Reaction

no.	*T* (K)	*k* (L mol^–1^ g^–1^ s^–1^)	*K*_A_(L mol^–1^)	*K*_B_(L mol^–1^)	*E* (kJ/mol)
1	343	0.211	0.042	0.018	41.2
2	353	0.329	0.041	0.015	
3	363	0.489	0.04	0.0149	
4	373	0.69	0.039	0.0141	
5	383	0.961	0.034	0.011	

Further, the pseudoequilibrium constants (*K*_E_′) were calculated for different parameters (Table 3). In the case of catalyst loading, the *K*_E_′ value was constant after 0.2 g, while in the case of the mole ratio, the *K*_E_′ values were almost constant, indicating that there is no effect of catalyst weight and mole ratio variation on the pseudoequilibrium constants. The same calculations were done for a different temperature, and the value of *K*_E_′ was found to increase with increasing temperature, which confirms that the pseudoequilibrium constant is only a function of the temperature, and thus, there is no mass transfer limitation.

**Table 3:** Pseudoequilibrium Constant (*K*′_E_) for Different Parameters

*K*_E_′	*w* (g)	*K*_E_′	mole ratio	*K*_E_′	*T* (K)
0.113555	0.05	0.549	1:01	0.313	343
0.275629	0.1	0.547	1:03	0.55	353
0.649102	0.2	0.549	1:05	0.93	363
0.636895	0.3	0.59	1:07	1.64	373
				1.99	383

### 3.5 Catalyst Reusability Studies

**Figure 10:**
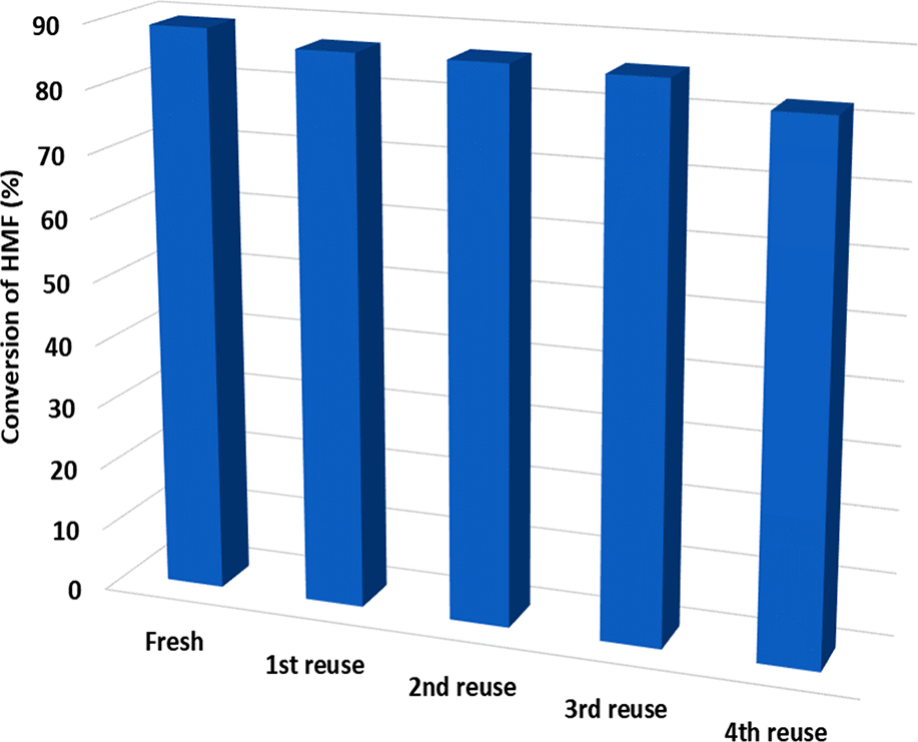
Catalyst reusability. Reaction conditions: HMF: 8.25 mmol, LA: 41.25 mmol, 20% w/w Sn_1_DTP/K-10 catalyst, catalyst weight: 0.2 g, speed of agitation: 1000 rpm, reaction time: 120 min.

The catalyst was separated by filtration, dried at 120 °C for 12 h, and used for the next reaction cycle. The catalyst was not regenerated. The yield of HMF levulinate decreased from 89.19% to 31.67% after 2 h. The reaction led to the formation of humins, which could be attributed to a partial blockage of the pores or active sites by the adsorbed species. Hence, the catalyst was regenerated by calcination at 550 °C for 3 h to remove adsorbed material on the catalyst surface, and the weight loss was made up with the fresh catalyst. The same procedure was repeated for up to four reaction cycles. The reaction shows a decrease in conversion of HMF to 82.37% for fourth cycle (Figure 10). TGA analysis of the reused catalyst shows that the catalyst is stable up to 300 °C.

A leaching test of the catalyst was performed by a hot filtration method to confirm the heterogeneous nature and stability of the prepared catalyst. The reaction was stopped after 30 min, as at this point 29.95% HMF converted to product. The catalyst was separated by filtration, and the clear reaction mass was run again at the same reaction conditions without catalyst. After 90 min, the reaction mass was analyzed, and it was found that there was no further conversion of the HMF observed. This confirms that there is no leaching of the catalyst in the reaction mass.

## 4 Conclusions

A novel efficient process for upgrading two important biomass molecules HMF and LA to HMF levulinate has been developed using Sn_1_-DTP/K-10 catalyst. High conversion of HMF, i.e., 90% within 2 h at 110 °C and catalyst loading of 0.2 g, confirms the high activity of the catalyst. The detailed characterization confirms the presence of a Keggin structure of DTP in supported forms. Further, the reaction parameters were optimized and the LHHW model was used to deduce the kinetic parameters. The rate constants were calculated at different temperatures and were used to make Arrhenius plots to get the activation energy for the reaction around 41.2 kJ/mol. This is the first attempt to predict the model for a given reaction. The catalyst is reusable for up to four cycles, and a hot filtration test indicated a heterogeneous nature of the catalytic activity.

## Nomenclature

*I*_S_: chemisorbed species
*C*_i_: concentration (mol/L)
*C*_t_: total concentration of the sites (mol/L)
*k*: reaction rate constant (L mol^–1^ g^–1^ s^–1^)
*K*: adsorption constant (L/mol)
*w*: catalyst loading (g/L)
*X*: fractional conversion
*K*_E_′: pseudoequilibrium constant
**Acronyms**
K-10: montmorillonite clay
DTP: Tungstophosphoric acid, i.e., H_3_PW_12_O_40_
Sn_1_-DTP/K10: Sn_1_ H_1_ PW_12_O_40_ supported on montmorillonite acid treated clay K-10
